# A Novel BRCA1 Pathogenic Variant in Tunisian Patient With High Grade Ovarian Cancer: Favorable Therapeutic Response to Olaparib

**DOI:** 10.1002/cnr2.70658

**Published:** 2026-09-03

**Authors:** Nihel Ammous‐Boukhris, Rania Abdelmaksoud‐Dammak, Ameni Feki, Souhir Khemiri, Slim Charfi, Tahia Sallemi‐Boudawara, Jamel Daoud, Afef Khanfir, Raja Mokdad Gargouri

**Affiliations:** ^1^ Center of Biotechnology of Sfax, Laboratory of Eukaryotes Molecular Biotechnology University of Sfax Sfax Tunisia; ^2^ Department of Medical Oncology Habib Bourguiba Hospital Sfax Tunisia; ^3^ Department of Anatomopathology Habib Bourguiba Hospital Sfax Tunisia; ^4^ Department of Radiotherapy Habib Bourguiba Hospital Sfax Tunisia

**Keywords:** *BRCA*, high grade serous ovarian cancer, next generation sequencing, pathogenic variant

## Abstract

**Background:**

Ovarian cancer is one of the leading causes of death from gynecological cancer worldwide. Genetic mutations in genes involved in key cellular functions such as *BRCA1/2* play a central role in tumorigenesis and have major implications for targeted therapeutic strategies, especially the use of poly (ADP‐ribose) polymerase (PARP) inhibitors.

**Case:**

Herein, we described a case of a 50‐year‐old woman diagnosed with severe anemia secondary to heavy menometrorrhagia. Initial gynecological evaluation, including transvaginal ultrasound, was unremarkable, and endometrial biopsy was not indicated. Imaging revealed no ovarian abnormalities; however, exploratory laparotomy identified a peritoneal nodule, leading to further investigation. Targeted NGS was performed on somatic and germline DNA samples and showed a frame shift deletion of 10 bp (c.1256_1265del: p.R419Ter) in the *BRCA1* gene. This variant, identified only in tumor tissues, is novel and classified as pathogenic in ClinVar and ACMG databases. Additional somatic alterations were detected in *TP53* and *MSH6*, while germline testing revealed only a variant of uncertain significance in *BARD1*. After first‐line chemotherapy, the patient benefited from olaparib and achieved a progression‐free survival of 23 months with good tolerance and no evidence of disease recurrence.

**Conclusion:**

This finding highlights the importance of integrating tumor‐based genomic profiling with germline testing to identify actionable mutations and guide precision oncology. The identification of a novel somatic *BRCA1* mutation expands the mutational spectrum of HGSOC and underscores the need to include underrepresented populations, such as those from North Africa, in genomic studies.

## 1 Introduction

Ovarian cancer (OC) is the second most common cause of gynecologic cancer death in women worldwide, largely due to its asymptomatic progression and diagnosis at advanced stages [1]. In Tunisia, the incidence of OC is 3.6 per 100 000 and accounts for approximately 1.6% of all cancer‐related deaths in women [2]. High Grade Serous Ovarian Cancer (HGSOC) is the most common type with aggressive biological behavior, high metastatic potential, and extensive genomic instability [3]. About 50% of HGSOC cases have a genetic mutation in DNA repair pathways, particularly in *BRCA1* and *BRCA2* genes, covering about 20%–25% of all HGSOC diagnosed [4, 5]. *BRCA*‐mutated tumors exhibit homologous recombination deficiency (HRD), rendering them more susceptible to therapeutic strategies that exploit synthetic lethality, especially poly (ADP‐ribose) polymerase (PARP) inhibitors (PARPi) [6]. PARPi act by trapping PARP enzymes at sites of DNA single‐strand breaks, resulting in replication‐dependent double‐strand breaks, which are normally repaired by the high‐fidelity process known as homologous recombination [7].

Dysfunctional homologous recombination in tumor cells, for example due to bi‐allelic loss of functional *BRCA1* or *BRCA2*, results in a reliance on error‐prone repair pathways, leading to an accumulation of DNA damage and ultimately cell death in tumor cells [8]. In the last decade, the therapeutic strategy for OC has evolved significantly with the introduction of PARPi that have demonstrated significant efficacy, especially when used as maintenance treatment of platinum‐sensitive recurrent ovarian cancer [9]. By targeting defective DNA repair mechanisms, PARPi induce genomic instability and selective tumor cell death.

Consequently, patients carrying pathogenic variants in *BRCA1*/*BRCA2* or other Homologous Recombination Repair (HRR) genes are more responsive to PARPi‐based therapies [10, 11].

Consequently, current clinical guidelines recommend systematic *BRCA* testing, including both germline and tumor‐based analyses, to identify patients who may benefit from targeted therapies.

In this context, we report a case of HGSOC in a Tunisian patient harboring a previously unreported somatic *BRCA1* mutation, highlighting the clinical relevance of comprehensive genomic profiling and contributing to the limited genomic data available from North African populations.

## 2 Case Description

A 50‐year‐old woman presented in 2022 to the Department of Surgery at CHU Habib Bourguiba of Sfax, Tunisia, with suspicion of a high‐grade serous carcinoma, likely of ovarian origin.

Seven months earlier, she had consulted for heavy menstrual bleeding and severe anemia. Her medical history included treated hypertension and she was premenopausal. Her surgical history included a cesarean section, cholecystectomy in 2010, and a previous ectopic pregnancy.

**FIGURE 1:**
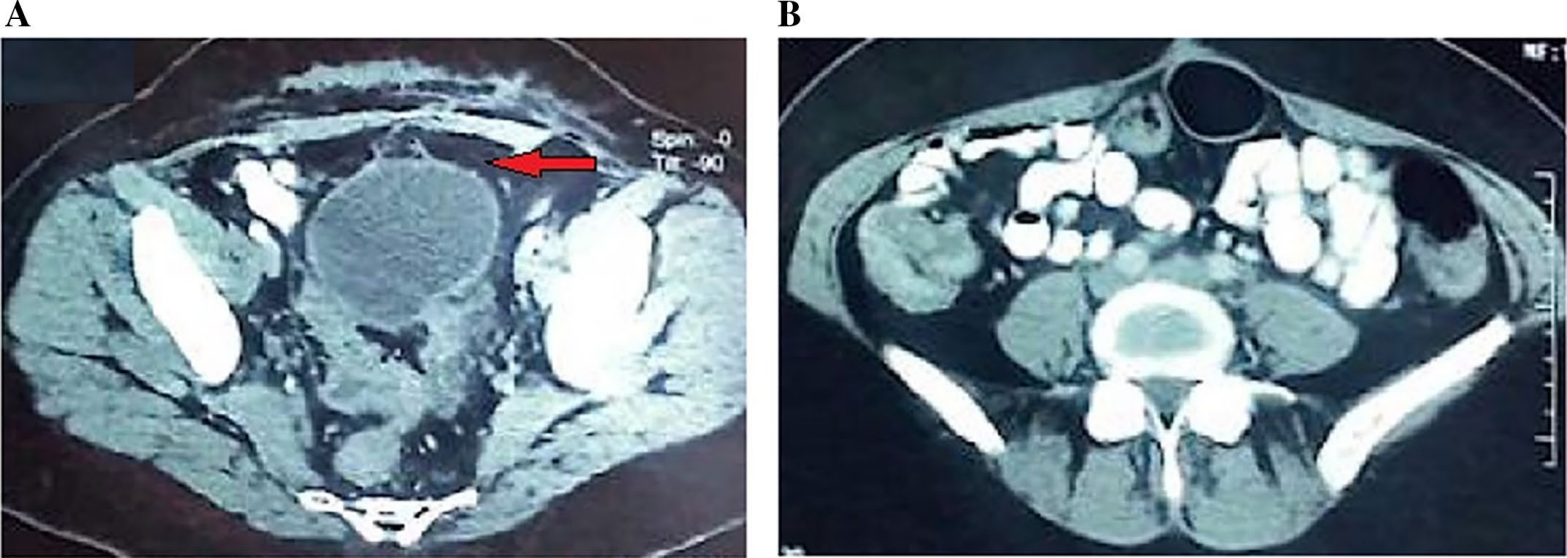
Contrast‐enhanced computed tomography (CT) images obtained before and after olaparib treatment. (A) Baseline CT scan showing the recurrent pelvic lesion (arrow) before initiation of olaparib. (B) Follow‐up CT scan demonstrating a significant reduction in lesion size after treatment.

Her family history revealed a paternal cousin who had lung cancer, breast cancer in a third‐degree relative, and a maternal grandmother who died of bladder cancer. On August 2022, she had an abdominopelvic CT scan showing that the uterus was enlarged with multiple fibroids and adenomyosis, while the ovaries were normal (Figure 1A). The patient underwent surgery a few days later. Intraoperative findings showed two peritoneal nodules beneath the liver, and the left adnexa was macroscopically normal. Complete resection of both peritoneal nodules, left adnexectomy, pelvic and aortic lymphadenectomi, and peritoneal cytology sampling were performed. Peritoneal biopsies of the paracolic gutters and resection of a different nodule were also performed.

Furthermore, the histopathological examination revealed the peritoneal infiltration with both peritoneal and retroperitoneal invasion. The mesosalpinx and ovarian parenchyma contained two small carcinomatous foci measuring 6 and 1 mm, respectively along with signs of vascular invasion. Endometriosis was also identified in the ovarian and mesosalpinx tissues; however, no direct causal relationship with HGSOC could be established. All lymph nodes were negative for metastasis.

**FIGURE 2:**
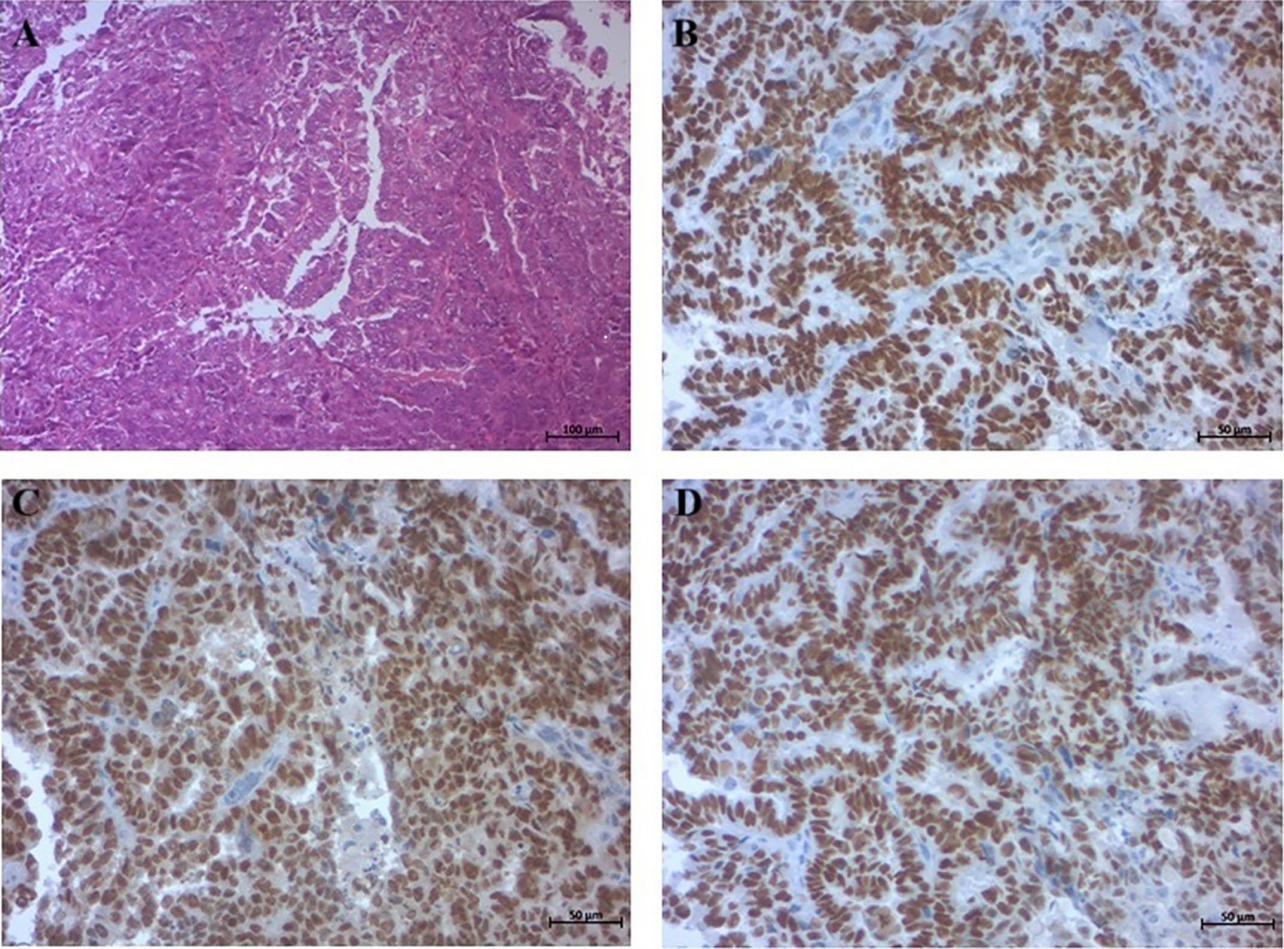
Histopathological and immunohistochemical characterization of the ovarian tumor. (A) Hematoxylin and eosin (H&E)‐stained section (original magnification ×100). Immunohistochemical analysis demonstrated diffuse nuclear positivity for PAX8 (B) (anti‐PAX8, clone IHC008, original magnification ×200), diffuse nuclear expression of WT1 (C) (anti‐WT1, clone WT49, original magnification ×200), and strong diffuse nuclear overexpression of p53 (D) (anti‐p53/TP53, clone DO‐7, original magnification ×200).

Immunohistochemical analysis confirmed the diagnosis of HGSOC based on positive expression of PAX8 and WT1 proteins (Figure 2B,C). Diffuse and strong nuclear expression of TP53 was also observed (Figure 2D).

The final pathological staging confirmed HGSOC, FIGO Stage III after complete surgical resection of the tumor. Tumor markers (CEA and CA‐125) were negative at baseline and remained within normal range throughout follow‐up. The patient subsequently received six cycles of adjuvant chemotherapy (paclitaxel 175 mg/m^2^ and carboplatin AUC 5 every 21 days between November 2022 and March 2023), achieving complete clinical and radiological response.

**FIGURE 3:**
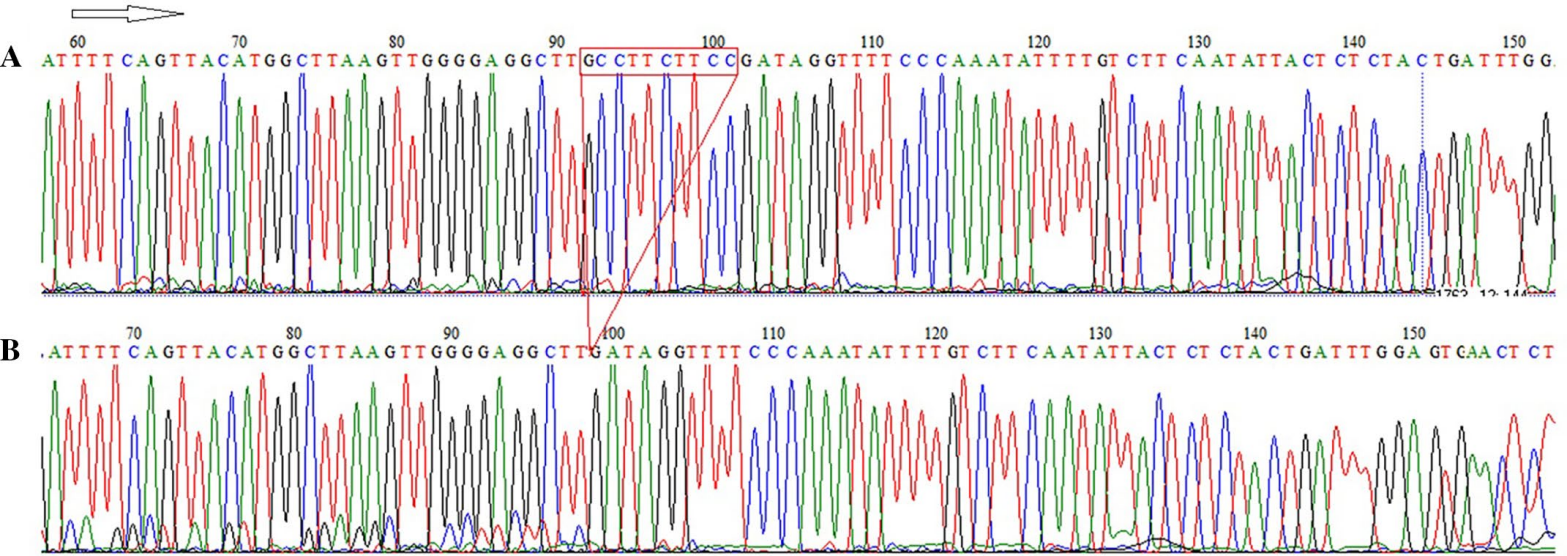
Sanger sequencing chromatograms of the BRCA1 gene from paired blood and tumor DNA. (A) DNA extracted from the patient's peripheral blood showing the wild‐type *BRCA1* sequence in exon 10, with no detectable sequence alteration. (B) DNA extracted from the tumor tissue demonstrating a 10‐base pair somatic deletion, c.1256_1265del (GCCTTCTTCC), in exon 10 of the *BRCA1* gene. The deleted nucleotides are highlighted by the red box, and the resulting shift in the chromatogram is indicated by the red arrow. The arrow indicated the sequencing direction.

To identify potential targets for personalized therapy, we performed Next Generation Sequencing (NGS) using a cancer panel including 31 cancer‐related genes on DNA extracted from both peripheral blood and formalin‐fixed paraffin‐embedded (FFPE) tumor samples. Libraries for targeted sequencing were prepared using the OncoRisk panel (Celemics, Seoul, Korea), covering 31 cancer‐associated genes. The detection of single‐nucleotide variants (SNVs) and insertions/deletions (INDELs) was generated using this panel that includes *BRCA1*, *BRCA2, PALB2, BARD1, BRIP1, RAD51C, RAD51D, RAD50, NBN, MRE11A, ATM, CHEK2, TP53, PTEN, APC, BLM, BMPR1A, CDH1, CDK4, CDKN2A, EPCAM, MLH1, MSH2, MSH6, MUTYH, PMS2, PRSS1, SLX4, SMAD4, STK11*, and *VHL*. Sequencing was performed on Illumina MiSeq Instrument (Illumina, San Diego, CA, USA) using 2 × 151 bp paired‐end reads on a V3 flow cell (Illumina). Raw sequencing reads were analyzed using the cloud‐based tool by illumina: basespace. Reads were aligned to the human reference genome (GRCh37/hg19), and variant calling was carried out on a panel of 31 genes that were selected based on their clinical actionability. During variant calling, modifications identified by the software were refined based on criteria including a coverage of more than 100X and a variant allele frequency (VAF) of at least 5%. The average sequencing depth was approximately 500X, with a minimum coverage threshold of 100X. Copy‐number analysis, formal LOH analysis, and detection of large genomic rearrangements were not included in the present workflow. After filtering, we retained 22 germline variants; most of them were classified as benign or likely benign, except a rare variant of uncertain significance (VUS) in the *BARD1* gene (NM_001282543): c.971C>T; p.(T324I). In tumor tissue, a frameshift *BRCA1* pathogenic variant (NM_007297): c.1256_1265del; p.(R419*) in exon 10 was identified. This variant has not been reported in ClinVar, COSMIC, or BIC databases at the time of analysis and it was detected only in tumor tissues confirming its somatic origin. Sanger sequencing confirmed the presence of the *BRCA1* c.1256_1265del variant in tumor DNA (Figure 3), and demonstrated an apparent absence of the wild‐type allele.

These findings are consistent with possible loss of heterozygosity; however, formal LOH and copy‐number analyses were not performed and therefore biallelic inactivation cannot be confirmed. Additional somatic pathogenic variants were detected in *TP53* (NM_001126118): c.380C>T; p.(S127F), and *MSH6* (NM_001281492): c.3601C>T; p.(R1201*) genes, along with several other VUS (Table S1). Variant allele frequencies for the reported variants were 48% (BRCA1), 74% (TP53), and 38% (MSH6).

Based on the efficacy of olaparib in the treatment of BRCA‐mutated advanced solid tumors, the patient received 300 mg of olaparib twice a day since May 24, 2023. The patient demonstrated a favorable response to olaparib, as evidenced by posttreatment CT imaging, normalization of CA‐125 levels, and a progression‐free survival (PFS) of 23 months, consistent with the established sensitivity of BRCA‐deficient tumors to PARPi.

## 3 Discussion

HGSOC is characterized by extensive genomic instability and frequent alterations affecting the homologous recombination DNA repair pathway. Among these, *BRCA1* and *BRCA2* mutations are the most clinically relevant, occurring in approximately 20%–25% of cases, with an additional proportion attributed to somatic mutations [4].

In this case, we identified a *previously unreported somatic BRCA1 frameshift mutation* (*c.1256_1265del; p.(Arg419*)), detected exclusively in tumor tissue. This alteration is predicted to produce a truncated protein, leading to loss of BRCA1 function and HRD, a key molecular hallmark of HGSOC.

*BRCA1* mutations, particularly those leading to loss of function, are well‐documented drivers of hereditary breast and ovarian cancer syndromes [4, 5]. While numerous *BRCA1* pathogenic variants have been reported globally, this case marks the first identification of a novel *BRCA1* mutation c.1256_1265del: p.R419* in a Tunisian patient diagnosed with HGSOC at the age of 50 years. This frameshift variant lies in the DNA binding domain resulting in a truncated BRCA1 protein of 466 aa compared with the full‐length protein of 1862 aa. Although the Sanger sequencing profile suggested possible loss of the wild‐type allele, formal LOH and copy‐number analyses were not available. Consequently, the presence of HRD was inferred from the truncating BRCA1 mutation but was not directly demonstrated, representing a limitation of the present study. The facts that this variant is not reported in the public databases, such as ClinVar, COSMIC, and BIC, and that it was only detected in tumor DNA, strongly support its classification as a pathogenic somatic variant. Such findings even emphasize the importance of incorporating heterogeneous underrepresented populations, such as North Africans, in genetic analysis and databases since population‐specific mutations may remain underreported. A significant advance in the treatment of *BRCA1*/2 mutated HGSOC has been achieved with the introduction of PARPi [9, 10, 11]. However, it was demonstrated that patients respond differently to PARPi depending on the type and location of *BRCA* pathogenic variant [12, 13, 14]. For instance, a recent study of Kim et al. investigated the PFS benefits of frontline PARPi therapy based on BRCA1/2 mutation locations. The authors showed that mutations within functional domains, particularly the DNA Binding Domain of BRCA1 and BRCA2, demonstrated the most pronounced benefit PARPi therapy [15]. Our is aligned with these observations, as the patient carrying a frameshift mutation within the BRCA1 DNA binding domain experienced a prolonged clinical response to olaparib, achieving a PFS of 23 months. Furthermore, it is important to note that in a recent analysis of the PAOLA‐1 Phase III clinical trial, researchers demonstrated that patients with *BRCA1*/2 mutations involving exon 11 derived greater benefit from olaparib plus bevacizumab compared with patients with mutations outside exon 11. Patients with mutations in the DNA‐BD of BRCA1 also showed significant benefit from olaparib plus bevacizumab [16, 17]. This suggests that the structural position of BRCA mutations plays an essential role in modulating treatment response which may be attributed to the presence of reversion mutation hotspots which is a recognized mechanism of resistance to PARPi. Notably, several studies reported that BRCA1 and BRCA2 DBD may be less prone to reversion mutations, potentially preserving HRD and thus maintaining the efficacy of PARPi [18, 19, 20].

The significance of the MSH6 variant c.3601C>T; p.(R1201*) is of uncertain functional significance in this case, given the absence of protein‐level or MSI testing.

The presence of a *TP53* mutation, observed in more than 95% of HGSOC cases, is consistent with the molecular profile described by The Cancer Genome Atlas (TCGA) [21]. From a pathological perspective, the identification of microscopic carcinomatous foci in macroscopically normal ovarian tissue was critical in establishing the diagnosis of primary ovarian cancer. This finding emphasizes the importance of thorough histopathological evaluation, particularly in atypical clinical presentations.

The coexistence of endometriosis in this case is noteworthy. Although endometriosis is well established as a precursor lesion for certain ovarian cancer subtypes, its role in HGSOC remains unclear. Some studies suggest that chronic inflammation and oxidative stress associated with endometriosis may contribute to tumorigenesis [22], although a direct causal relationship cannot be established in this case.

Lastly, this case highlights the complexity of ovarian cancer's mutational landscape and underlines the value of comprehensive genomic profiling. Identifying a pathogenic *BRCA1* mutation has implications not only for patient management, but also for risk assessment among her family members, who may benefit from predictive testing and preventive strategies.

Nevertheless, several limitations should be acknowledged, including the single‐case nature of this report and the absence of HRD analysis and the lack of functional validation of the identified variant.

## 4 Conclusion

This report highlights the relevance of genetic testing in guiding personalized treatment for ovarian cancer patients. The identification of a novel *BRCA1* mutation emphasizes the clinical utility of tumor‐based genomic profiling.

These findings support the integration of next‐generation sequencing into routine clinical practice and underscore the importance of expanding genomic studies in underrepresented populations to improve precision oncology strategies.

## Author Contributions

**Souhir Khemiri:** conceptualization, writing – review and editing. **Nihel Ammous‐Boukhris:** conceptualization, methodology, formal analysis, writing – original draft, writing – review and editing. **Slim Charfi:** conceptualization, methodology, writing – review and editing. **Tahia Sallemi‐Boudawara:** formal analysis, supervision, writing – review and editing, conceptualization. **Ameni Feki:** conceptualization, writing – review and editing. **Jamel Daoud:** conceptualization, supervision, writing – review and editing. **Rania Abdelmaksoud‐Dammak:** conceptualization, methodology, writing – review and editing. **Raja Mokdad Gargouri:** investigation, formal analysis, supervision, writing – review and editing, writing – original draft. **Afef Khanfir:** conceptualization, supervision, writing – review and editing.

## Funding

This work was supported by Tunisian Ministry of higher education and scientific research (LR19CBS02).

## Ethics Statement

All samples were handled in compliance with the Declaration of Helsinki.

## Consent

The patient signed a written informed consent for publication.

## Conflicts of Interest

The authors declare no conflicts of interest.

## Supporting information


**Table S1:** List of germinal and somatic variants identified in the patient.

## Data Availability

The novel genetic variant identified in this study has been submitted to the ClinVar database (accession number: VCV004849514.1). All relevant data supporting the findings of this case report are included within the article and its Supporting Information. Additional details are available from the corresponding author upon reasonable request.

## References

[1] F. Bray , M. Laversanne , H. Sung , et al., “Global Cancer Statistics 2022: GLOBOCAN Estimates of Incidence and Mortality Worldwide for 36 Cancers in 185 Countries,” CA: A Cancer Journal for Clinicians 74, no. 3 (2024): 229–263, 10.3322/caac.21834. 38572751

[2] Z. Momenimovahed , A. Tiznobaik , S. Taheri , and H. Salehiniya , “Ovarian Cancer in the World: Epidemiology and Risk Factors,” International Journal of Women's Health 11 (2019): 287–299, 10.2147/IJWH.S197604. PMC6500433 31118829

[3] A. Gurung , T. Hung , J. Morin , and C. B. J. Gilks , “Molecular Abnormalities in Ovarian Carcinoma: Clinical, Morphological and Therapeutic Correlates,” Histopathology 62, no. 1 (2013): 59–70, 10.1111/his.12033. 23240670

[4] B. M. Norquist , M. I. Harrell , M. F. Brady , et al., “Inherited Mutations in Women With Ovarian Carcinoma,” JAMA Oncology 2, no. 4 (2016): 482–490, 10.1001/jamaoncol.2015.5495. 26720728 PMC4845939

[5] S. Zhang , R. Royer , S. Li , et al., “Frequencies of BRCA1 and BRCA2 Mutations Among 1,342 Unselected Patients With Invasive Ovarian Cancer,” Gynecologic Oncology 121, no. 2 (2011): 353–357, 10.1016/j.ygyno.2011.01.020. 21324516

[6] W. G. Kaelin , “The Concept of Synthetic Lethality in the Context of Anticancer Therapy,” Nature Reviews. Cancer 5, no. 9 (2005): 689–698, 10.1038/nrc1691. 16110319

[7] A. J. Cortez , P. Tudrej , K. A. Kujawa , and K. M. Lisowska , “Advances in Ovarian Cancer Therapy,” Cancer Chemotherapy and Pharmacology 81, no. 1 (2018): 17–38, 10.1007/s00280-017-3501-8. 29249039 PMC5754410

[8] M. E. Gee , Z. Faraahi , A. McCormick , and R. J. Edmondson , “DNA Damage Repair in Ovarian Cancer: Unlocking the Heterogeneity,” Journal of Ovarian Research 11, no. 1 (2018): 50, 10.1186/s13048-018-0424. 29925418 PMC6011341

[9] M. Javle and N. J. Curtin , “The Role of PARP in DNA Repair and Its Therapeutic Exploitation,” British Journal of Cancer 105, no. 8 (2011): 1114–1122, 10.1038/bjc.2011.382. 21989215 PMC3208503

[10] D. M. O'Malley , T. C. Krivak , N. Kabil , et al., “PARP Inhibitors in Ovarian Cancer: A Review,” Targeted Oncology 18, no. 4 (2023): 471–503, 10.1007/s11523-023-00970-w. 37268756 PMC10344972

[11] S. Polajžer and K. Černe , “Precision Medicine in High‐Grade Serous Ovarian Cancer: Targeted Therapies and the Challenge of Chemoresistance,” International Journal of Molecular Sciences 26, no. 6 (2025): 2545, 10.3390/ijms26062545. 40141188 PMC11942020

[12] V. Tuninetti , J. A. Marín‐Jiménez , G. Valabrega , and E. Ghisoni , “Long‐Term Outcomes of PARP Inhibitors in Ovarian Cancer: Survival, Adverse Events, and Post‐Progression Insights,” ESMO Open 9, no. 11 (2024): 103984, 10.1016/j.esmoop.2024.103984. 39541620 PMC11613435

[13] W. P. Tew , C. Lacchetti , A. Ellis , et al., “PARP Inhibitors in the Management of Ovarian Cancer: ASCO Guideline,” Journal of Clinical Oncology 38, no. 30 (2020): 3468–3493, 10.1200/JCO.20.01924. 32790492 PMC8942301

[14] C. Marchetti , A. Fagotti , R. Fruscio , et al., “Benefit From Maintenance With PARP Inhibitor in Newly Diagnosed Ovarian Cancer According to BRCA1/2 Mutation Type and Site: A Multicenter Real‐World Study,” ESMO Open 10, no. 4 (2025): 104533, 10.1016/j.esmoop.2025.104533. 40174507 PMC11999263

[15] J. H. Kim , S. I. Kim , E. T. Kim , et al., “The Association Between Location of BRCA Mutation and Efficacy of PARP Inhibitor as a Frontline Maintenance Therapy in Advanced Epithelial Ovarian Cancer,” Cancers 17, no. 5 (2025): 756, 10.3390/cancers17050756. 40075604 PMC11899539

[16] I. Ray‐Coquard , P. Pautier , S. Pignata , et al., “Olaparib Plus Bevacizumab as First‐Line Maintenance in Ovarian Cancer,” New England Journal of Medicine 381, no. 25 (2019): 2416–2428, 10.1056/NEJMoa1911361. 31851799

[17] P. Harter , M. A. Mouret‐Reynier , S. Pignata , et al., “Efficacy of Maintenance Olaparib Plus Bevacizumab According to Clinical Risk in Patients With Newly Diagnosed, Advanced Ovarian Cancer in the Phase III PAOLA‐1/ENGOT‐ov25 Trial,” Gynecologic Oncology 164, no. 2 (2022): 254–264, 10.1016/j.ygyno.2021.12.016. 34952708

[18] N. Cordani , T. Bianchi , L. C. Ammoni , et al., “An Overview of PARP Resistance in Ovarian Cancer From a Molecular and Clinical Perspective,” International Journal of Molecular Sciences 24, no. 15 (2023): 11890, 10.3390/ijms241511890. 37569269 PMC10418869

[19] L. Collet , B. Hanvic , M. Turinetto , et al., “BRCA1/2 Alterations and Reversion Mutations in the Area of PARP Inhibitors in High Grade Ovarian Cancer: State of the Art and Forthcoming Challenges,” Frontiers in Oncology 14 (2024): 1354427, 10.3389/fonc.2024.1354427. 38544832 PMC10965616

[20] K. Nakamura , H. Hayashi , R. Kawano , et al., “BRCA1/2 Reversion Mutations in a Pan‐Cancer Cohort,” Cancer Science 11, no. 2 (2024): 635–647, 10.1111/cas.16033. PMC10859608 38041241

[21] The Cancer Genome Atlas Research Network , “Integrated Genomic Analyses of Ovarian Carcinoma,” Nature 474 (2011): 609–615, 10.1038/nature10166. 21720365 PMC3163504

[22] M. S. Anglesio , N. Papadopoulos , A. Ayhan , et al., “Cancer‐Associated Mutations in Endometriosis Without Cancer,” New England Journal of Medicine 376 (2017): 1835–1848, 10.1056/NEJMoa1614814. 28489996 PMC5555376

